# Association Between Parental Education and Simultaneous Malnutrition Among Parents and Children in 45 Low- and Middle-Income Countries

**DOI:** 10.1001/jamanetworkopen.2022.51727

**Published:** 2023-01-24

**Authors:** Shaoru Chen, Sol Richardson, Yuhao Kong, Ning Ma, Ai Zhao, Yi Song, Chunling Lu, S. V. Subramanian, Zhihui Li

**Affiliations:** 1 Vanke School of Public Health, Tsinghua University, Beijing, China; 2 Institute for Healthy China, Tsinghua University, Beijing, China; 3 Institute of Child and Adolescent Health, School of Public Health, Peking University, Beijing, China; 4 National Health Commission Key Laboratory of Reproductive Health, Peking University, Beijing, China; 5 Department of Global Health and Social Medicine, Harvard Medical School, Boston, Massachusetts; 6 Division of Global Health Equity, Brigham and Women’s Hospital, Boston, Massachusetts; 7 Harvard Center for Population and Development Studies, Cambridge, Massachusetts; 8 Department of Social and Behavioral Sciences, Harvard T. H. Chan School of Public Health, Boston, Massachusetts

## Abstract

**Importance:**

Parental education is known to be associated with the health status of parents and their offspring. However, the association between parental education and the simultaneous manifestation of multiple forms of malnutrition within households remains underinvestigated globally.

**Objective:**

To assess the association between parental education and the simultaneous manifestation of malnutrition of both parent and child (either overnutrition or undernutrition)—referred to as the double burden of malnutrition (DBM)—at the household level in mother-child and father-child pairs in low- and middle-income countries (LMICs).

**Design, Setting, and Participants:**

This cross-sectional study used data from the US Agency for International Development Demographic and Health Surveys (January 1, 2010, to December 31, 2021) to identify mother-child pairs and father-child pairs from LMICs. The eligibility criteria were as follows: (1) children aged 0 to 59 months; (2) nonpregnant mothers at the time of the survey in the sample of mother-child pairs; and (3) valid measures of the weight, height, and hemoglobin level for the child and at least 1 of their parents.

**Exposures:**

Highest level of parental education obtained and number of years of education completed.

**Main Outcomes and Measures:**

Four sets of multivariable logistic regression models were constructed to assess the association between parental education and DBM, and analysis was performed between March 10 and May 15, 2022.

**Results:**

This study included 423 340 mother-child pairs from 45 LMICs and 56 720 father-child pairs from 16 LMICs. The mean (SD) age of the mother-child pairs was 28.2 (6.1) and 1.9 (1.4) years, respectively; 48.8% of the children were female. We observed that 49.0% of mother-child pairs experienced DBM. Compared with mother-child pairs with no maternal education, higher maternal education was associated with a lower risk of DBM. For example, the odds ratio (OR) for tertiary maternal education was 0.71 (95% CI, 0.67-0.74). However, the association differed by DBM subtypes: higher maternal education was associated with a lower risk of both mothers and children being undernourished but with a higher risk of almost all DBM subtypes involving overnutrition. For example, compared with mother-child pairs with no maternal education, those with secondary education were less likely to develop simultaneous maternal and child undernutrition (OR, 0.83 [95% CI, 0.80-0.86]) but were more likely to experience simultaneous maternal and child overnutrition (OR, 2.20 [95% CI, 1.61-3.00]); similar results were observed for pairs with primary and tertiary education. The results in mother-child pairs remained consistent after controlling for paternal education. Among the father-child pairs, 26.5% had DBM, with fathers with tertiary education significantly more likely to experience simultaneous paternal overnutrition and child undernutrition (OR, 1.55 [95% CI, 1.23-1.95]) compared with pairs with no paternal education; they were also less likely to have both paternal and child undernutrition (OR, 0.70 [95% CI, 0.59-0.84]).

**Conclusions and Relevance:**

In this study, maternal education and paternal education were independently associated with DBM, and the associations differed by DBM subtypes. These findings suggest that the different risks of malnutrition faced by households with various levels of education should thus be considered in policy evaluation.

## Key Points

**Question:**

What is the association between parental education and the simultaneous manifestation of malnutrition of both parent and child—referred to as the double burden of malnutrition (DBM)—within households in low- and middle-income countries?

**Findings:**

This cross-sectional study of 423 340 mother-child pairs and 56 720 father-child pairs from low- and middle-income countries found that parental education was associated with the risk of DBM, but the associations differed by DBM subtypes. More advanced parental education was associated with a higher risk of DBM subtypes involving overnutrition (eg, maternal overnutrition and child undernutrition) and a lower risk of undernutrition for both the parent and child.

**Meaning:**

These findings highlight the association between parental education and household nutritional status, suggesting that policymakers should differentiate subgroups when formulating future policies.

## Teaser

This cross-sectional study uses international survey data to examine the association between parental education and the simultaneous manifestation of malnutrition of both parent and child in households in low- and middle-income countries.

## Introduction

Ending all forms of malnutrition by 2030 is at the top of the global health agenda during the United Nations’ sustainable development goals (SDG) era.^1^ Low- and middle-income countries (LMICs) are prioritized, given their high prevalence of stunting, underweight, and micronutrient deficiencies.^2^ Despite much progress made during the past decades to reduce undernutrition, the rising issue of obesity and overweight in LMICs has led to profound concerns over the simultaneous manifestation of multiple forms of malnutrition, requiring multifactorial action to address the problem.^3^

The double burden of malnutrition (DBM) is increasingly acknowledged as a new challenge to achieving the SDG targets to end malnutrition.^3^ The DBM, which usually refers to the coexistence of overnutrition and undernutrition, can be measured at the individual, household, community, and country level.^3^ The DBM at the household level (referring to ≥1 individuals with overnutrition or undernutrition within the same household) is of great policy importance because households are the most essential units affecting the dietary patterns, eating behaviors, and food choices of household members.^4^ Indeed, DBM exists in 2% to 35% of households in LMICs; in countries such as Guatemala, Egypt, and Comoros, more than 20% of households experience this problem.^3^ However, the means to identify households at high risk of DBM in LMICs has been underinvestigated.

Maternal education has frequently been examined as a risk factor for a single form of malnutrition such as child stunting, wasting, and underweight.^2,5,6^ In terms of studies on DBM, maternal education has often been included as a covariate, but the findings have been inconsistent. Some studies reported that higher maternal education was associated with a higher risk of DBM.^7,8,9^ A study in Myanmar found that households with mothers with primary education had 62% higher odds of experiencing DBM than those with no maternal education.^7^ Conversely, other studies found that higher maternal education was associated with a lower risk of DBM.^10,11,12^ For example, a study in South and Southeast Asia suggested that households with mothers with primary education were 16% more likely to experience DBM than households with mothers with secondary or higher education.^10^ However, other studies reported no association between maternal education and DBM.^9,13,14^ The lack of consensus might be attributed to the varying contexts and relatively small sample sizes of these studies. To date, studies have not taken a global perspective on the role of maternal education in influencing the issue of DBM.

Furthermore, most previous studies have focused on mother-child pairs. The education level of fathers, who often act as household heads and decision-makers within the household, may be a factor in their health and the health of their children and other household members.^5,6,15^ In a study analyzing the data of 7662 mother-child pairs in Bangladesh, investigators reported an association between higher paternal education and a lower risk of DBM.^16^ However, the influence of paternal education on household-level DBM in father-child pairs has been rarely investigated.^17^

Using the most recent data from the US Agency for International Development Demographic and Household Surveys (DHS), we aimed to explore the association between parental education and DBM in both mother-child and father-child pairs. Furthermore, we examined whether the associations differed by various DBM subtypes.

## Methods

### Data Sources

This cross-sectional study was approved by the Tsinghua Institutional Review Board. Informed consent was waived because publicly available data were used. The study followed the Strengthening the Reporting of Observational Studies in Epidemiology (STROBE) reporting guideline.

We conducted cross-sectional analyses using the most recent data from the DHS conducted between January 1, 2010, to December 31, 2021. These large-scale, nationally representative household health surveys use a stratified multistage sampling design.^18^ The cross-sectional surveys collect demographic, socioeconomic, and health information on children and their parents using a complete standardized questionnaire. As surveys from earlier years may be inconsistent in data collection and measurement design, we excluded surveys earlier than 2010 to ensure the quality of this study.^19^

### Study Population and Sample Size

The eligibility criteria for our analytic sample were as follows: (1) children aged 0 to 59 months; (2) nonpregnant mothers at the time of the survey in the sample of mother-child pairs; and (3) valid measures of weight, height, and hemoglobin level for the child and at least 1 of their parents. We identified 423 340 mother-child pairs from 45 countries and 56 720 father-child pairs from 16 countries for primary analysis in our cross-sectional study. The flowcharts and countries included are presented in eFigures 1 and 2 and eTables 1 and 2 in Supplement 1, respectively.

### Outcome Measures

The main outcomes of our study were DBM and all DBM subtypes. The DBM in mother-child pairs was defined as the mother and their offspring within a family simultaneously having at least 1 form of malnutrition. Similarly, DBM in father-child pairs was defined as the simultaneous manifestation of paternal and child malnutrition. In our study, we followed previous practice and classified DBM into the following 4 subtypes: (1) coexistence of maternal (paternal) overnutrition and child undernutrition, (2) coexistence of maternal (paternal) undernutrition and child overnutrition, (3) coexistence of maternal (paternal) overnutrition and child overnutrition, and (4) coexistence of maternal (paternal) undernutrition and child undernutrition.^3,7,20^

We constructed dichotomous variables for DBM and all DBM subtypes, with 1 indicating the households confronting adverse health outcomes and 0 indicating otherwise. For children, overnutrition referred to a weight-for-age *z* score of more than 2 SDs; undernutrition referred to 1 or more of the following conditions: underweight (weight-for-age *z* score of <−2 SD), wasting (weight-for-height *z* score of <−2 SD), stunting (height-for-age *z* score of <−2 SD), or anemia (hemoglobin count <11 g/dL).^21^ For adults, overnutrition was defined as a body mass index (BMI; calculated as weight in kilograms divided by height in meters squared) of 25 or greater; undernutrition was defined as having 1 or more of the following conditions: underweight (BMI <18.5), short stature (mother’s height <145 cm; father’s height <155 cm), or anemia (hemoglobin count <12 g/dL).^2^

### Exposures and Covariates

We measured parental education using the highest level of education obtained and the number of years of education completed. We constructed a categorical variable for each parent as follows: (1) no education, (2) primary education, (3) secondary education, and (4) tertiary education. Covariates included child factors (age, birth order, sex, and birth size), household factors (type of residence, household wealth quintile, and sex of household head), and environmental factors (indoor pollution, drinking water source, and sanitary facility).^2^ Definitions of the covariates are listed in eTable 3 in Supplement 1. The child’s age was considered as a continuous variable in the main analyses and as a categorical variable (<1 year and 1-5 years) in the stratified analysis. For mother-child pairs, we also included maternal factors (mother’s age, skilled birth attendant at delivery, family planning needs satisfied, and maternal age at marriage). For the father-child pairs, we considered child factors, household factors, and paternal factors, including the father’s age and age at marriage (eTable 3 in Supplement 1).

### Statistical Analysis

We assessed the association between parental education and DBM by pooling data from all countries. To ensure that the estimates were representative in pooled analyses, we not only took into account the sample weights, clustering, and stratification variables provided by the DHS, we also reweighted the observations according to each country’s population size.^2,22^ We clustered the sample at the level of the primary sampling unit, which allowed for interdependence of error terms within clusters and households. We included country-fixed effects to account for the unobservable country-level factors.

We conducted 4 sets of multivariable logistic regression models to calculate the odds ratios (ORs) and corresponding 95% CIs for the prevalence of DBM subtypes by education level. First, we adjusted country-fixed effects in model 1 and then adjusted for child factors, maternal age at the time of the survey, and household factors in model 2. We further adjusted for maternal factors and sex of household head in model 3 and additionally adjusted for environmental factors in model 4. We examined the possible nonlinear association between parental education and DBM using nonparametrically restricted cubic splines, in which the number of years of education completed was used to measure parental education. In the main analyses, we did not adjust for parent education level.

Furthermore, to avoid the potential impact of subgroup analyses on type I error, we applied the method of Bonferroni correction. We conducted stratified analyses and effect modifications by children’s age, sex, and type of residence to investigate whether the associations differed by various subgroups. In addition, we grouped countries according to the World Bank income classification to explore the associations among countries with different income levels.^23^

We conducted sensitivity analyses to test the robustness of the results. First, to investigate whether maternal education was associated with DBM independent of paternal education, we included paternal education in the model. Similarly, we also added sensitivity analyses adjusting for maternal education when examining the association between paternal education and DBM. Second, observations with missing data for key covariates were dropped from our pooled analysis in order to show results without imputation. Third, for households with more than 1 child with malnutrition, we only used data for the oldest child and their father or mother in order to avoid overcounting. Fourth, although we reweighted the observations by country population size, we conducted a sensitivity analysis that excluded India to mitigate its large sample size. Finally, we performed a sensitivity analysis leaving birth size out as a covariate to rule out low birth size as a factor.

We used Stata, version 17 (StataCorp LLC) for all analyses. All descriptive analyses used 2-sided tests. As in previous studies,^2,24^ we dropped pairs with missing outcomes or exposures. The percentages of missing values of covariates were less than 4%. We performed multiple imputation for these missing values.^2,25^ Missing values of key covariates were imputed based on child sex, residence, household wealth quintile, and sex of the household head. Imputations were performed using logistic regression for categorical covariates, using 5 multiple imputation data sets. Values with *P* < .05 were considered statistically significant. The analysis was performed between March 10 and May 15, 2022.

## Results

In this study, we identified 423 340 mother-child pairs from 45 countries in the DHS data. The mean (SD) age of the mother-child pairs was 28.2 (6.1) years and 1.9 (1.4) years, respectively. There were 207 573 mother-child pairs (49.0%) with DBM, 177 083 (41.8%) with simultaneous maternal and child undernutrition, and 56 020 (13.5%) with maternal overnutrition and child undernutrition (Table 1). The prevalence of DBM was lower for pairs with mothers with more education than for mothers with no education. Among all 4 DBM subtypes, the 3 involving overnutrition were more likely to occur in mother-child pairs with higher maternal education. For example, the prevalence of simultaneous maternal overnutrition and child undernutrition was 18.5% among the pairs with mothers with tertiary education compared with 10.4% for mothers with no education. The prevalence of simultaneous maternal undernutrition and child undernutrition was higher among pairs with mothers with no education (47.9%) than for mothers with tertiary education (33.8%) (Table 1).

**Table 1.** Characteristics of Mother-Child Pairs Across 45 Low- and Middle-Income Countries by Maternal Education Level

Characteristic	No. of mother-child pairs (%)					*P* value
	Overall (n = 423 340)	Maternal education level^a^				
		None (n = 111 457)	Primary (n = 100 203)	Secondary (n = 169 515)	Tertiary (n = 42 165)	
**Children**						
Age, y, mean (SD)	1.9 (1.4)	2 (1.4)	1.9 (1.4)	1.9 (1.4)	1.9 (1.4)	<.001
Birth order, mean (SD)	2.8 (2.0)	3.8 (2.3)	3.2 (2.1)	2.1 (1.3)	1.7 (0.9)	<.001
Sex						
Male	216 761 (51.2)	56 615 (50.8)	50 847 (50.7)	87 228 (51.5)	22 071 (52.3)	<.001
Female	206 579 (48.8)	54 842 (49.2)	49 356 (49.3)	82 287 (48.5)	20 094 (47.7)	
Birth size						
Average	258 760 (61.1)	63 787 (57.2)	56 006 (55.9)	109 565 (64.6)	29 402 (69.7)	<.001
Smaller than average	55 124 (13.0)	16 787 (15.1)	14 845 (14.8)	19 451 (11.5)	4041 (9.6)	
Larger than average	102 397 (24.2)	27 718 (24.9)	27 445 (27.4)	38 636 (22.8)	8598 (20.4)	
Missing	7059 (1.7)	3165 (2.8)	1907 (1.9)	1863 (1.1)	124 (0.3)	
Household						
Type of residence						
Urban	111 933 (26.4)	17 400 (15.6)	22 498 (22.5)	51 733 (30.5)	20 302 (48.2)	<.001
Rural	311 407 (73.6)	94 057 (84.4)	77 705 (77.5)	117 782 (69.5)	21 863 (51.8)	
Wealth quintile						
Lowest	111 202 (26.3)	48 137 (43.2)	32 494 (32.4)	28 795 (17.0)	1776 (4.2)	<.001
Lower	95 314 (22.5)	27 558 (24.7)	26 098 (26.1)	37 630 (22.2)	4028 (9.6)	
Middle	82 816 (19.6)	18 067 (16.2)	19 888 (19.9)	38 126 (22.5)	6735 (16.0)	
Higher	74 348 (17.6)	12 363 (11.1)	14 203 (14.2)	36 931 (21.8)	10 851 (25.7)	
Highest	59 660 (14.0)	5332 (4.8)	7520 (7.4)	28 033 (16.5)	18 775 (44.5)	
Sex of household head						
Male	346 250 (81.8)	92 820 (83.3)	79 370 (79.2)	138 827 (81.9)	35 233 (83.6)	<.001
Female	77 087 (18.2)	18 635 (16.7)	20 833 (20.8)	30 687 (18.1)	6932 (16.4)	
Environment						
Drinking water source						
Unsafe	69 240 (16.4)	27 211 (24.4)	24 489 (24.4)	16 053 (9.5)	1487 (3.5)	<.001
Safe	340 214 (80.4)	82 205 (73.8)	73 252 (73.1)	146 703 (86.5)	38 054 (90.3)	
Missing	13 886 (3.2)	2041 (1.8)	2462 (2.5)	6759 (4.0)	2624 (6.2)	
Sanitary facility						
Not improved	148 082 (35.0)	63 690 (57.2)	43 408 (43.3)	38 072 (22.5)	2912 (6.9)	<.001
Improved	261 462 (61.8)	45 722 (41.0)	54 385 (54.3)	124 725 (73.6)	36 630 (86.9)	
Missing	13 796 (3.2)	2045 (1.8)	2410 (2.4)	6718 (4.0)	2623 (6.2)	
Indoor pollution						
Low	130 853 (30.9)	12 934 (11.6)	15 526 (15.5)	72 799 (43.0)	29 594 (70.2)	<.001
High	278 568 (65.8)	96 451 (86.5)	82 243 (82.1)	89 938 (53.1)	9936 (23.5)	
Missing	13 919 (3.3)	2072 (1.9)	2434 (2.4)	6778 (4.0)	2635 (6.3)	
**Mothers**						
Age, y, mean (SD)	28.2 (6.1)	29.9 (6.7)	28.5 (6.6)	26.8 (5.3)	28.8 (4.8)	<.001
Skilled birth attendant at delivery						
No	89 630 (21.2)	42 421 (38.1)	27 331 (27.3)	18 402 (10.9)	1476 (3.5)	<.001
Yes	323 543 (76.4)	63 998 (57.4)	69 031 (68.9)	150 083 (88.5)	40 431 (95.9)	
Missing	10 167 (2.4)	5038 (4.5)	3841 (3.8)	1030 (0.6)	258 (0.6)	
Family planning needs satisfied						
No	98 022 (23.2)	30 131 (27.0)	24 617 (24.5)	35 039 (20.7)	8235 (19.5)	<.001
Yes	323 300 (76.3)	80 809 (72.5)	75 215 (75.1)	133 551 (78.8)	33 725 (80.0)	
Missing	2018 (0.5)	517 (0.5)	371 (0.4)	925 (0.5)	205 (0.5)	
Maternal age at marriage, y						
<18	259 043 (61.2)	53 404 (47.9)	51 010 (50.9)	115 596 (68.2)	39 033 (92.6)	<.001
≥18	153 513 (36.3)	57 038 (51.2)	45 614 (45.5)	48 244 (28.5)	2617 (6.2)	
Missing	10 784 (2.5)	1015 (0.9)	3579 (3.6)	5675 (3.3)	515 (1.2)	
**Mother-child pair nutrition status**						
DBM						
No	215 767 (51.0)	52 084 (46.7)	56 317 (56.2)	83 708 (49.4)	23 658 (56.1)	<.001
Yes	207 573 (49.0)	59 373 (53.3)	43 886 (43.8)	85 807 (50.6)	18 507 (43.9)	
Maternal overnutrition and child undernutrition^b^						
No	358 012 (86.5)	98 695 (89.6)	82 698 (86.4)	142 586 (85.7)	34 033 (81.5)	<.001
Yes	56 020 (13.5)	11 469 (10.4)	12 997 (13.6)	23 840 (14.3)	7714 (18.5)	
Maternal undernutrition and child overnutrition^b^						
No	414 026 (99.4)	109 846 (99.6)	99 134 (99.6)	164 953 (99.3)	40 093 (99.1)	<.001
Yes	2341 (0.6)	486 (0.4)	378 (0.4)	1123 (0.7)	354 (0.9)	
Maternal overnutrition and child overnutrition^b^						
No	405 294 (99.5)	108 872 (99.8)	94 670 (99.6)	162 153 (99.5)	39 599 (98.9)	<.001
Yes	1836 (0.5)	188 (0.2)	349 (0.4)	860 (0.5)	439 (1.1)	
Maternal undernutrition and child undernutrition						
No	246 257 (58.2)	58 120 (52.1)	64 052 (63.9)	96 154 (56.7)	27 931 (66.2)	<.001
Yes	177 083 (41.8)	53 337 (47.9)	36 151 (36.1)	73 361 (43.3)	14 234 (33.8)	

Abbreviation: DBM, double burden of malnutrition.

^a^ Education level refers to maternal education level in the study of mother-child pairs.

^b^ Sample sizes for DBM subtypes were as follows: 414 032 for maternal overnutrition and child undernutrition, 416 367 for maternal undernutrition and child overnutrition, and 407 130 for maternal overnutrition and child overnutrition.

Our sample also included 56 720 father-child pairs from 16 countries (Table 2), among which 15 057 had DBM (26.5%). The prevalence of DBM was higher among pairs with fathers with more education than for fathers with no education (Table 2). In terms of DBM subtypes, father-child pairs with higher paternal education were more likely to have any form of DBM involving overnutrition and both the father and child were less likely to be undernourished.

**Table 2.** Characteristics of Father-Child Pairs Across 16 Low- and Middle-Income Countries by Parental Education Level

Characteristic	No. of father-child pairs (%)					*P* value
	Overall (n = 56 720)	Paternal education level^a^				
		None (n = 14 377)	Primary (n = 12 065)	Secondary (n = 24 395)	Tertiary (n = 5883)	
**Children**						
Age, y, mean (SD)	2.0 (1.4)	2.0 (1.4)	2.0 (1.4)	2.0 (1.4)	1.9 (1.4)	<.001
Birth order, mean (SD)	3.1 (2.2)	4.0 (2.4)	3.5 (2.3)	2.6 (1.8)	2.0 (1.4)	<.001
Sex						
Male	29 033 (51.2)	7365 (51.2)	6103 (50.6)	12 485 (51.2)	3080 (52.4)	.17
Female	27 687 (48.8)	7012 (48.8)	5962 (49.4)	11 910 (48.8)	2803 (47.6)	
Birth size						
Average	32 984 (58.2)	8014 (55.7)	6580 (54.5)	14 579 (59.8)	3811 (64.8)	<.001
Smaller than average	6959 (12.3)	2030 (14.1)	1617 (13.5)	2720 (11.2)	592 (10.1)	
Larger than average	15 552 (27.3)	3877 (27.0)	3525 (29.2)	6716 (27.5)	1434 (24.4)	
Missing	1225 (2.2)	456 (3.2)	343 (2.8)	380 (1.5)	46 (0.7)	
Household						
Type of residence						
Urban	14 287 (25.2)	2081 (14.5)	2248 (18.6)	7163 (29.4)	2795 (47.5)	<.001
Rural	42 433 (74.8)	12 296 (85.5)	9817 (81.4)	17 232 (70.6)	3088 (52.5)	
Wealth quintile						
Lowest	14 700 (25.9)	5300 (36.9)	4303 (35.7)	4832 (19.8)	265 (4.5)	<.001
Lower	12 690 (22.4)	3417 (23.8)	3072 (25.5)	5587 (22.9)	614 (10.4)	
Middle	11 179 (19.7)	2728 (19.0)	2278 (18.8)	5356 (22.0)	817 (13.9)	
Higher	10 371 (18.3)	2038 (14.2)	1612 (13.4)	5109 (20.9)	1612 (27.4)	
Highest	7780 (13.7)	894 (6.1)	800 (6.6)	3511 (14.4)	2575 (43.8)	
Sex of household head						
Male	52 890 (93.2)	13 833 (96.2)	11 395 (94.5)	22 316 (91.5)	5346 (90.9)	<.001
Female	3830 (6.8)	544 (3.8)	670 (5.6)	2079 (8.5)	537 (9.1)	
Environment						
Drinking water source						
Unsafe	12 273 (21.6)	4144 (28.8)	3386 (28.1)	4346 (17.8)	397 (6.8)	<.001
Safe	44 067 (77.7)	10 201 (71.0)	8587 (71.1)	19 854 (81.4)	5425 (92.2)	
Missing	380 (0.7)	32 (0.2)	92 (0.8)	195 (0.8)	61 (1.0)	
Sanitary facility						
Not improved	25 684 (45.3)	10 068 (70.0)	6861 (56.8)	8025 (32.9)	730 (12.4)	<.001
Improved	30 719 (54.2)	4279 (29.8)	5138 (42.6)	16 208 (66.4)	5094 (86.6)	
Missing	317 (0.5)	30 (0.2)	66 (0.6)	162 (0.7)	59 (1.0)	
Indoor pollution						
Low	14 831 (26.2)	1037 (7.2)	1703 (14.1)	8590 (35.2)	3501 (59.5)	<.001
High	41 547 (73.3)	13 299 (92.5)	10 284 (85.2)	15 641 (64.1)	2323 (39.5)	
Missing	342 (0.5)	41 (0.3)	78 (0.7)	164 (0.7)	59 (1.0)	
**Fathers**						
Age, y, mean (SD)	34.7 (7.8)	38 (8.7)	34.6 (7.8)	33.1 (7.1)	33.8 (6.5)	<.001
Family planning needs satisfied						
No	13 556 (23.9)	3943 (27.4)	3300 (27.4)	5222 (21.4)	1091 (18.5)	<.001
Yes	43 122 (76.0)	10 420 (72.5)	8758 (72.6)	19 153 (78.5)	4791 (81.5)	
Missing	42 (0.1)	14 (0.1)	7 (0.0)	20 (0.1)	1 (0.0)	
Paternal age at marriage, y						
<18	51 142 (90.2)	12 975 (90.3)	10 514 (87.1)	22 111 (90.6)	5542 (94.2)	<.001
≥18	3798 (6.7)	1020 (7.0)	1152 (9.6)	1486 (6.1)	140 (2.4)	
Missing	1780 (3.1)	382 (2.7)	399 (3.3)	798 (3.3)	201 (3.4)	
**Father-child pair nutrition status**						
DBM						
No	41 663 (73.5)	11 189 (77.8)	9383 (77.8)	17 035 (69.8)	4056 (68.9)	<.001
Yes	15 057 (26.5)	3188 (22.2)	2682 (22.2)	7360 (30.2)	1827 (31.1)	
Paternal overnutrition and child undernutrition^b^						
No	31 670 (85.6)	5259 (90.9)	6837 (90.3)	15 546 (83.8)	4028 (78.8)	<.001
Yes	5335 (14.4)	524 (9.1)	733 (9.7)	2995 (16.2)	1083 (21.2)	
Paternal undernutrition and child overnutrition^b^						
No	56 271 (99.7)	14 297 (99.9)	11 995 (99.8)	24 156 (99.7)	5823 (99.7)	.003
Yes	148 (0.3)	20 (0.1)	28 (0.2)	81 (0.3)	19 (0.3)	
Paternal overnutrition and child overnutrition^b^						
No	36 561 (99.5)	5729 (99.9)	7499 (99.5)	18 303 (99.5)	5030 (99.1)	<.001
Yes	188 (0.5)	8 (0.1)	38 (0.5)	98 (0.5)	44 (0.9)	
Paternal undernutrition and child undernutrition						
No	45 828 (80.8)	11 524 (80.2)	9988 (82.8)	19 370 (79.4)	4946 (84.1)	<.001
Yes	10 892 (19.2)	2853 (19.8)	2077 (17.2)	5025 (20.6)	937 (15.9)	

Abbreviation: DBM, double burden of malnutrition.

^a^ Education level refers to paternal education level in the study of father-child pairs.

^b^ Sample sizes for DBM subtypes were as follows: 37 005 for paternal overnutrition and child undernutrition, 56 419 for paternal undernutrition and child overnutrition, and 36 749 for paternal overnutrition and child overnutrition.

### Associations Between Maternal Education and DBM in Mother-Child Pairs

Table 3 shows the associations between maternal education and DBM. In general, the mother-child pairs with higher maternal education were less likely to have DBM. For example, compared with the pairs with no maternal education, those with tertiary education showed lower odds of having DBM (OR, 0.71 [95% CI, 0.67-0.74]). The results of models 1 through 3 were generally consistent with the results of model 4 (Table 3).

**Table 3.** Association Between Parental Education Level and Double Burden of Malnutrition Subtypes^a^

Nutrition status	Odds ratio (95% CI)			
	Model 1^b^	Model 2^c^	Model 3^d^	Model 4^e^
**Mother-child pairs across 45 LMICs**				
DBM				
No education	1 [Reference]	1 [Reference]	1 [Reference]	1 [Reference]
Primary education	0.88 (0.85-0.91)	0.94 (0.90-0.97)	0.94 (0.90-0.97)	0.94 (0.91-0.97)
Secondary education	0.75 (0.72-0.77)	0.88 (0.85-0.91)	0.88 (0.85-0.91)	0.89 (0.86-0.92)
Tertiary education	0.52 (0.50-0.55)	0.69 (0.66-0.73)	0.70 (0.66-0.74)	0.71 (0.67-0.74)
Maternal overnutrition and child undernutrition				
No education	1 [Reference]	1 [Reference]	1 [Reference]	1 [Reference]
Primary education	1.37 (1.29-1.46)	1.28 (1.20-1.36)	1.27 (1.19-1.35)	1.26 (1.19-1.34)
Secondary education	1.76 (1.67-1.85)	1.38 (1.31-1.46)	1.37 (1.30-1.45)	1.35 (1.28-1.43)
Tertiary education	2.55 (2.40-2.72)	1.36 (1.26-1.46)	1.36 (1.26-1.46)	1.33 (1.24-1.43)
Maternal undernutrition and child overnutrition				
No education	1 [Reference]	1 [Reference]	1 [Reference]	1 [Reference]
Primary education	0.84 (0.67-1.04)	0.79 (0.63-0.98)	0.79 (0.63-0.98)	0.78 (0.63-0.98)
Secondary education	1.39 (1.17-1.64)	1.12 (0.95-1.34)	1.12 (0.94-1.34)	1.11 (0.93-1.33)
Tertiary education	1.79 (1.45-2.22)	1.14 (0.89-1.46)	1.13 (0.88-1.46)	1.12 (0.87-1.44)
Maternal overnutrition and child overnutrition				
No education	1 [Reference]	1 [Reference]	1 [Reference]	1 [Reference]
Primary education	2.23 (1.57-3.15)	1.80 (1.28-2.55)	1.75 (1.24-2.47)	1.74 (1.23-2.46)
Secondary education	4.26 (3.17-5.72)	2.35 (1.72-3.21)	2.25 (1.64-3.07)	2.20 (1.61-3.00)
Tertiary education	10.02 (7.25-13.85)	3.21 (2.21-4.66)	3.10 (2.12-4.53)	3.01 (2.07-4.39)
Maternal undernutrition and child undernutrition				
No education	1 [Reference]	1 [Reference]	1 [Reference]	1 [Reference]
Primary education	0.84 (0.81-0.87)	0.91 (0.87-0.94)	0.91 (0.88-0.94)	0.91 (0.88-0.95)
Secondary education	0.65 (0.63-0.68)	0.81 (0.79-0.84)	0.82 (0.79-0.85)	0.83 (0.80-0.86)
Tertiary education	0.39 (0.37-0.42)	0.63 (0.60-0.67)	0.64 (0.60-0.67)	0.64 (0.61-0.68)
**Father-child pairs across 16 LMICs**				
DBM in father-child pairs				
No education	1 [Reference]	1 [Reference]	1 [Reference]	1 [Reference]
Primary education	0.82 (0.72-0.93)	0.87 (0.76-0.99)	0.87 (0.76-0.99)	0.87 (0.76-0.99)
Secondary education	0.81 (0.73-0.91)	0.93 (0.83-1.05)	0.94 (0.83-1.05)	0.94 (0.83-1.06)
Tertiary education	0.77 (0.67-0.88)	0.93 (0.80-1.08)	0.93 (0.80-1.08)	0.93 (0.80-1.08)
Paternal overnutrition and child undernutrition				
No education	1 [Reference]	1 [Reference]	1 [Reference]	1 [Reference]
Primary education	1.21 (0.97-1.51)	1.12 (0.89-1.40)	1.12 (0.89-1.40)	1.12 (0.89-1.40)
Secondary education	1.96 (1.63-2.35)	1.37 (1.13-1.67)	1.37 (1.12-1.67)	1.37 (1.13-1.68)
Tertiary education	2.95 (2.40-3.62)	1.55 (1.23-1.94)	1.54 (1.23-1.94)	1.55 (1.23-1.95)
Paternal undernutrition and child undernutrition				
No education	1 [Reference]	1 [Reference]	1 [Reference]	1 [Reference]
Primary education	0.75 (0.65-0.85)	0.82 (0.72-0.94)	0.82 (0.72-0.94)	0.83 (0.72-0.95)
Secondary education	0.58 (0.51-0.65)	0.82 (0.72-0.92)	0.82 (0.73-0.92)	0.82 (0.73-0.93)
Tertiary education	0.36 (0.31-0.42)	0.69 (0.58-0.83)	0.70 (0.59-0.83)	0.70 (0.59-0.84)

Abbreviations: DBM, double burden of malnutrition; LMIC, low- to middle-income country.

^a^ Education level refers to maternal education level in the study of mother-child pairs and paternal education level in the study of father-child pairs.

^b^ Adjusted country-fixed effects.

^c^ Additionally adjusted for child age, child birth order, maternal age, child sex, child birth size, type of residence, and household wealth quintile.

^d^ Additionally adjusted for skilled birth attendant at delivery, family planning needs satisfied, maternal age at marriage, and sex of household head.

^e^ Additionally adjusted for indoor pollution, drinking water source, and sanitary facility.

When examining the DBM subtypes, we found that more advanced maternal education was associated with a higher risk of experiencing the DBM subtypes involving overnutrition. For example, compared with mother-child pairs with no maternal education, those with secondary maternal education were more likely to experience simultaneous maternal overnutrition and child undernutrition (OR, 1.35 [95% CI, 1.28-1.43]) as well as simultaneous maternal and child overnutrition (OR, 2.20 [95% CI, 1.61-3.00]). In terms of simultaneous maternal undernutrition and child overnutrition, although associations were found for secondary education (OR, 1.39 [95% CI, 1.17-1.64]) and tertiary education (OR, 1.79 [95% CI, 1.45-2.22]), the statistical significance disappeared after covariate adjustment, with ORs of 1.11 (95% CI, 0.93-1.13) and 1.12 (95% CI, 0.87-1.44), respectively.

**Figure 1.**
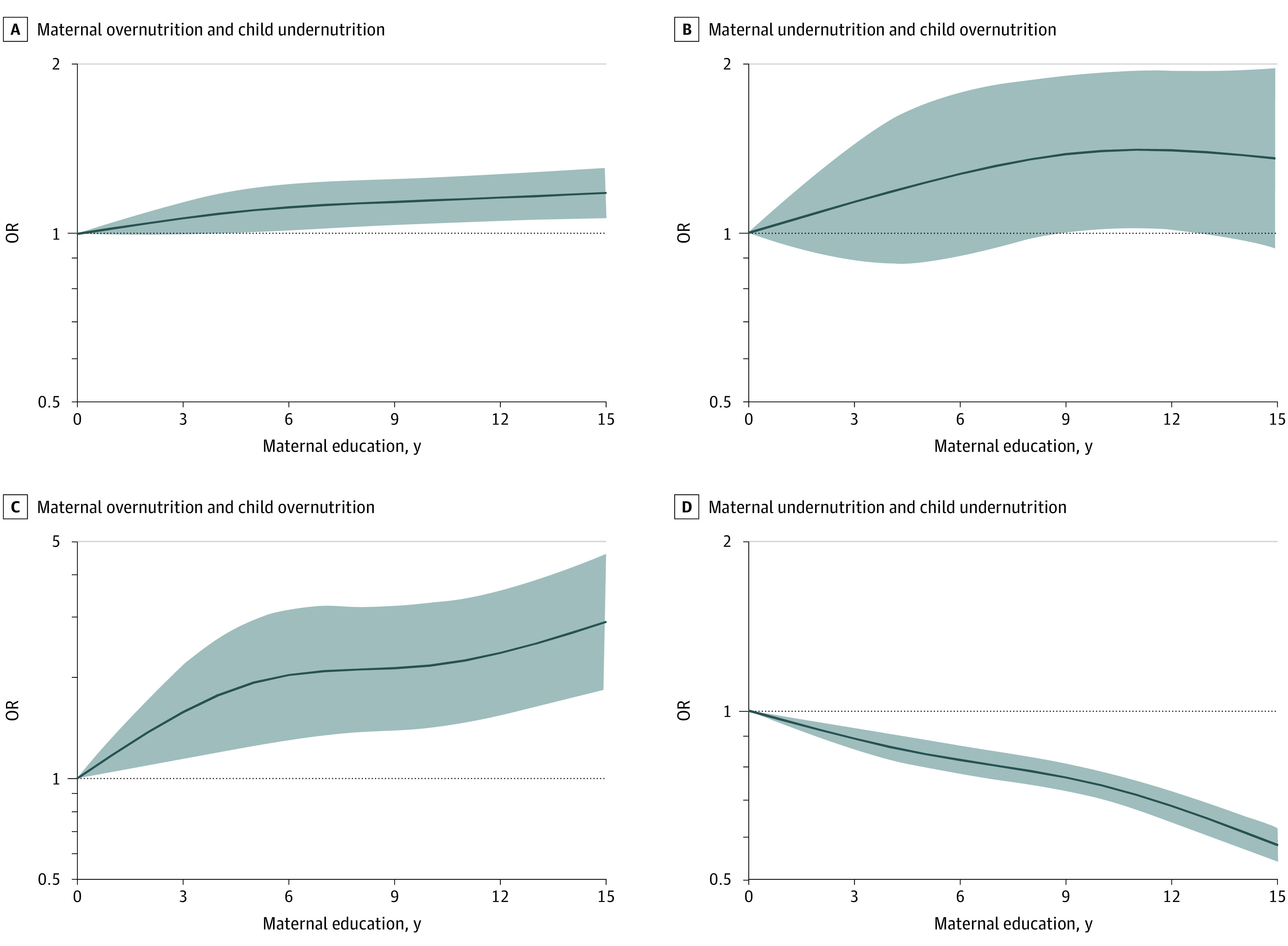
Dose-Response Association Between Years of Maternal Education and Prevalence of Double Burden of Malnutrition Subtypes A, Maternal overnutrition and child undernutrition. B, Maternal undernutrition and child overnutrition. C, Maternal overnutrition and child overnutrition. D, Maternal undernutrition and child undernutrition. Associations were examined with logistic regression models based on restricted cubic splines. Solid lines represent estimates of odds ratios (ORs) and shaded areas represent 95% CIs.

Moreover, we found that a higher level of maternal education was associated with a lower risk of simultaneous maternal and child undernutrition. Compared with the pairs with no maternal education, those with tertiary education had a 36% lower risk of undernutrition in both the mother and child (OR, 0.64 [95% CI, 0.61-0.68]) (Table 3). The dose response of maternal education years is shown in Figure 1 and eFigure 3 in Supplement 1. These results were consistent with the main analyses.

**Figure 2.**
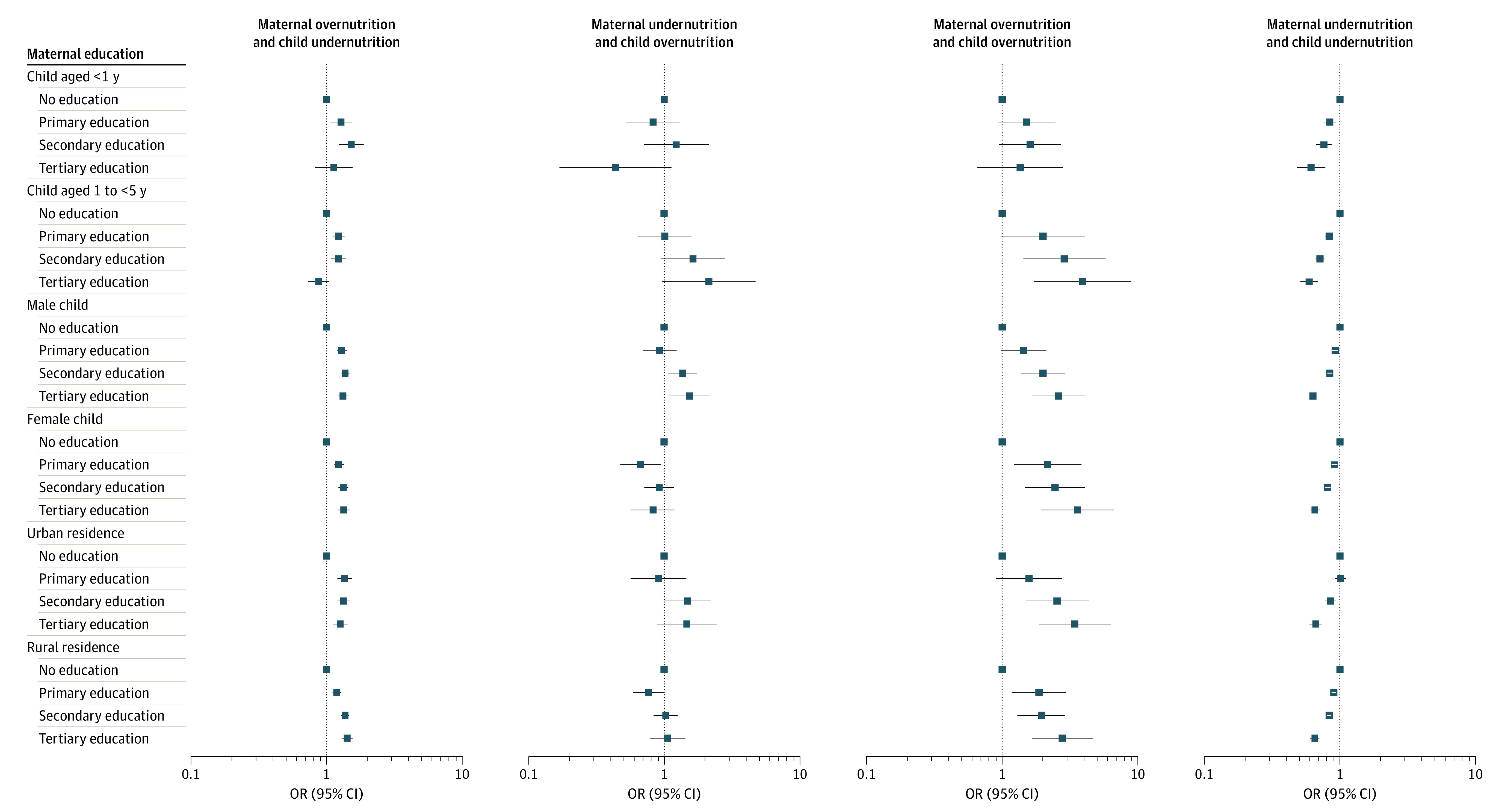
Association Between Maternal Education Level and Double Burden of Malnutrition Subtypes Stratified by Child Age, Sex, and Type of Residence Odds ratios (ORs) and 95% CIs were estimated using logistic regression adjusting for child birth order, maternal age, child birth size, household wealth quintile, skilled birth attendant at delivery, family planning needs satisfied, maternal age at marriage, sex of household head, indoor pollution, drinking water source, sanitary facility, and country-fixed effects.

Results of stratified analyses by children’s age, sex, and type of residence are presented in Figure 2 and eFigure 4 in Supplement 1. The results were similar to the main analyses. The interaction terms were not significant for children’s sex and type of residence. However, we found significant interactions (eTable 4 in Supplement 1) with children’s age, with associations among pairs of children aged between 1 and 5 years compared with those aged younger than 1 year. For example, secondary maternal education was associated with a higher risk of DBM among children aged between 1 and 5 years (OR, 2.88 [95% CI, 1.43-5.79]); however, no such association was found among children aged younger than 1 year (OR, 1.61 [95% CI, 0.95-2.74]).

We generated stratified analyses by country income classification, and the results are presented in eTable 5 in Supplement 1. We found an association between maternal education and DBM among low- and lower-middle-income countries; despite the relatively large magnitude, no association was found among upper-middle-income countries. For example, compared with the pairs with no maternal education, pairs with mothers with tertiary education were more likely to have DBM, with ORs of 0.62 (95% CI, 0.55-0.71) in low-income countries, 0.72 (95% CI, 0.68-0.77) in lower-middle-income countries, and 1.09 (95% CI, 0.52-2.29) in upper-middle-income countries.

### Associations Between Paternal Education and DBM in Father-Child Pairs

No association was found between paternal education and DBM in general (Table 3). Due to sample-size limitations, we only analyzed 2 DBM subtypes: (1) coexistence of paternal undernutrition and child overnutrition and (2) coexistence of paternal and child undernutrition. Similar to maternal education, we found that compared with households with no paternal education, households with fathers with secondary or tertiary education were more likely to have simultaneous paternal overnutrition and child undernutrition, with ORs of 1.37 (95% CI, 1.13-1.68) and 1.55 (95% CI, 1.23-1.95), respectively. The pairs with fathers with higher education were less likely to have simultaneous paternal and child undernutrition (Table 3), and associations were found for secondary education (OR, 0.82 [95% CI, 0.73-0.93]) and tertiary education (OR, 0.70 [95% CI, 0.59-0.84]) . Dose-response results were consistent with the main analyses (eFigure 5 in Supplement 1). In the stratified analyses (eFigure 6 in Supplement 1), there were no associations between paternal education and DBM prevalence for most subgroups.

### Sensitivity Analysis

After further adjusting for paternal education as a covariate in our analysis in the mother-child pairs, the association remained statistically significant (eTable 6 in Supplement 1). For example, before and after adjusting for paternal education level, pairs with mothers with tertiary education were more likely to have DBM, with ORs of 0.71 (95% CI, 0.67-0.74) and 0.68 (95% CI, 0.61-0.75), respectively. The results also stayed unchanged in sensitivity analyses after dropping observations with missing values (eTable 7 in Supplement 1), using data for the oldest children only (eTable 8 in Supplement 1), excluding India (eTable 9 in Supplement 1), or leaving birth size out as a covariate (eTable 10 in Supplement 1). The results of the sensitivity analyses on paternal education were consistent with the main analyses and are presented in eTables 11 through 13 in Supplement 1. For example, fathers with tertiary education were significantly more likely to have simultaneous paternal overnutrition and child undernutrition (OR, 1.46 [95% CI, 1.14-1.87]) compared with pairs with no paternal education, and they were less likely to have both paternal and child undernutrition (OR, 0.81 [95% CI, 0.68-0.98]) even after adjusting for maternal education (eTable 11 in Supplement 1).

## Discussion

This cross-sectional study of parental education and household DBM in LMICs had 3 salient findings. First, we observed that higher maternal education was associated with a lower risk of DBM in mother-child pairs. Second, the associations between maternal education and DBM differed by DBM subtypes. Higher maternal education was associated with a higher risk of having DBM subtypes involving overnutrition and a lower risk of both the mother and child being undernourished. Even after adjusting for paternal education in mother-child pairs, the results remained consistent. Third, more advanced paternal education was associated with a higher risk of simultaneous paternal overnutrition and child undernutrition but with a lower risk of simultaneous undernutrition in both the father and the child, especially in pairs with fathers with tertiary education. The association of paternal education was independent of maternal education.

Our findings on the association between maternal education and DBM subtypes involving overnutrition are supported by previous evidence.^7,8,9^ Previous studies have shown that higher education is associated with higher odds of obesity in households, especially in some LMICs.^26,27^ At the individual level, women with more education are more likely to have jobs with higher wages, increasing the household income and indirectly leading to better nutrition levels among household members.^5^ In turn, children of mothers with more education are more likely to be sedentary and consequently overweight.^26^ At the societal level, cultural and societal perceptions may drive some people to be obese as it is considered healthy.^28^ In some LMICs, high-calorie foods are expensive and more likely to be served in urban settings where wealthy households reside.^29^ Low physical activity levels and consumption of high-calorie Western foods are perceived as status symbols in some countries.^30^ For example, in Nigeria, cultural traditions encourage women to consume high-calorie foods to gain weight before marriage.^28^

We also observed that paternal education was associated with DBM subtypes even after adjusting for maternal education. Some previous studies support our finding regarding paternal education and child malnutrition.^5,6^ Our results suggest that the association of maternal and paternal education level with child nutrition was independent and that the association was greater for maternal education level. However, some studies have found that fathers with higher levels of education were also more likely to have spouses with higher levels of education,^31^ which may complicate comparisons of the association of maternal and paternal education with regard to children’s health outcomes. Our study indicated a different finding on the associations between paternal education and DBM subtypes. With the development of the SDGs, the implementation of supportive policies in many countries, and significant progress toward achieving gender equality in education access and attainment, paternal education is more likely to play a key role in improving child health, especially in patrilineal countries.^32^

Household-level DBM has been defined in different ways according to the World Health Organization (WHO) and other research and reports. For example, Sunuwar et al^33^ defined the pair with maternal overnutrition and child anemia as a household with DBM. The WHO has traditionally defined DBM as the coexistence of undernutrition and overweight/obesity^34^; yet in some cases, the WHO also includes the coexistence of maternal anemia and child undernutrition as a subtype of DBM.^35^ In our study, we included 4 DBM subtypes; 2 fit the traditional WHO definition, including the coexistence of maternal/paternal overnutrition and child undernutrition as well as the coexistence of maternal/paternal undernutrition and child overnutrition. Regarding the 2 traditional WHO-defined subtypes, we observed an association between parental education and the occurrence of DBM, suggesting that households with parents with more education were more likely to have various household members experiencing undernutrition and overnutrition simultaneously. Our finding was different from previous studies, mostly based on the context of developed settings such as the United Kingdom and Germany, showing that more advanced education was a protective factor for both underweight and overweight.^36,37^ Our results suggest that in LMICs, higher parental education is a risk factor for all DBM subtypes involving overnutrition, including the traditional WHO-defined DBM subtypes, which might be attributed to the nutrition transition in LMICs.^3^

We consider maternal short stature as a form of maternal undernutrition based on the United Nations Children’s Fund definition of maternal malnutrition,^38^ which is also used widely in other studies.^39,40^ Studies have shown that maternal short stature is associated with a higher risk of having children with undernutrition, as it may lead to an inadequate supply of nutrients to the fetus and, consequently, affect the child’s health.^41^ Mothers with short stature might have a greater need for education on healthy eating and a healthy lifestyle to prevent transgenerational undernutrition.

Eliminating malnutrition has been at the top of the global health agenda for decades. Although numerous attempts have been made at different levels (eg, community, local, national, etc.) worldwide, most were targeted at a single form of malnutrition such as child stunting, obesity, or iron deficiency.^42^ There is increasing evidence showing that interventions targeting a single form of malnutrition could lead to unexpected outcomes. For example, a study in Peru reported that food assistance programs were associated with a lower risk of obesity in children and a higher risk of obesity in mothers.^43^ Policies targeting household-level DBM should therefore recognize that addressing one form of malnutrition did not exacerbate other types of malnutrition.^42,44^ Education is generally associated with good nutrition and is key for addressing all forms of malnutrition. Our findings suggest that policy makers should differentiate between subgroups and develop targeted interventions. In terms of policy making, more attention is needed to address overnutrition among families with higher education.

### Limitations

This cross-sectional study has several limitations. First, the use of observational and cross-sectional data hampered our ability to make any causal inferences in our study. Second, although the magnitude of our sample was large, we did not find any association between maternal education and DBM among upper-middle-income countries, probably due to the limited number of countries in this category (only 6 countries). Third, although our study showed an association between parental education and DBM, the mechanisms through which parental education influences DBM are not fully understood and need to be further investigated. Finally, because of the large number of missing values for anthropometric indicators of fathers in the DHS, only 16 countries were included in the study of father-child pairs; therefore, studies of DBM in father-child pairs need to be further explored.

## Conclusions

The association we observed between parental education and DBM differed by specific malnutrition type, with a higher level of parental education associated with a higher risk of having almost all DBM subtypes involving overnutrition and with a lower risk of both the parent and child being undernourished. This finding has important implications for policymakers to differentiate between subgroups and develop targeted interventions. Our results suggest that greater attention on the situation of overnutrition among families with higher education is needed when formulating policy. Nutrition education should be conducted with parents with more education to reduce DBM subtypes involving overnutrition. For families with parents with less education, we suggest that it is important to improve maternal education levels and provide nutrition education for parents to reduce the DBM of undernutrition.

## Notes

Supplement 1. **eTable 1.** Countries Included in the Analysis of Mother-Child Pairs **eTable 2.** Countries Included in the Analysis of Father-Child Pairs **eTable 3.** Definitions of Covariates **eTable 4.** *P* Values of Tests of Interaction Terms From Logistic Regression Models for the Subgroup Analysis **eTable 5.** Association Between Maternal Education Level and Double Burden of Malnutrition Subtypes in Different Income Groups (Odds Ratios [95% CIs]) **eTable 6.** Association Between Maternal Education Level and Double Burden of Malnutrition Subtypes in Different Income Groups, Further Adjusting for Paternal Education Level (Odds Ratios [95% CIs]) **eTable 7.** Association Between Maternal Education Level and Double Burden of Malnutrition Subtypes Without Using Multiple Imputation (Odds Ratios [95% CIs]) **eTable 8.** Association Between Maternal Education Level and Double Burden of Malnutrition Subtypes in Mothers With the Oldest Child (Odds Ratios [95% CIs]) **eTable 9.** Association Between Maternal Education Level and Double Burden of Malnutrition Subtypes, Further Excluding India (Odds Ratios [95% CIs]) **eTable 10.** Association Between Maternal Education Level and Double Burden of Malnutrition Subtypes, Without Adjusting for Birth Size (Odds Ratios [95% CIs]) **eTable 11.** Association Between Paternal Education Level and Double Burden of Malnutrition Subtypes, Further Adjusting for Maternal Education Level (Odds Ratios [95% CIs]) **eTable 12.** Association Between Paternal Education Level and Double Burden of Malnutrition Subtypes Without Using Multiple Imputation (Odds Ratios [95% CIs]) **eTable 13.** Association Between Paternal Education Level and Double Burden of Malnutrition Subtypes in Fathers With the Oldest Child (Odds Ratios [95% CIs]) **eFigure 1.** Flow Diagram Showing Exclusions and Final Sample Sizes of the Study Population in the Study of Mother-Child Pairs, Using the Most Recent Pooled Demographic and Household Surveys Data Since 2010 **eFigure 2.** Flow Diagram Showing Exclusions and Final Sample Sizes of the Study Population in the Study of Father-Child Pairs, Using the Most Recent Pooled Demographic and Household Surveys Data Since 2010 **eFigure 3.** Dose-Response Association Between Years of Maternal Education and Prevalence of Double Burden of Malnutrition **eFigure 4.** Associations Between Maternal Education Level and Double Burden of Malnutrition Stratified by Child’s Age, Sex, and Residence (Odds Ratios [95% CIs]) **eFigure 5.** Dose-Response Association Between Years of Paternal Education and Prevalence of Double Burden of Malnutrition Subtypes **eFigure 6.** Associations Between Paternal Education Level and Double Burden of Malnutrition Subtypes Stratified by Child’s Age, Sex, and Type of Residence (Odds Ratios [95% CIs])

Supplement 2.
Data Sharing Statement
