# Supplementary material for: Association Between Parental Education and Simultaneous Malnutrition Among Parents and Children in 45 Low- and Middle-Income Countries
Source: JAMA Netw Open. 2023 Jan 24;6(1):e2251727. doi: 10.1001/jamanetworkopen.2022.51727 (PMC10408270; doi:10.1001/jamanetworkopen.2022.51727)
Supplement: Supplement 1. — eTable 1. Countries Included in the Analysis of Mother-Child Pairs eTable 2. Countries Included in the Analysis of Father-Child Pairs eTable 3. Definitions of Covariates eTable 4. P Values of Tests of Interaction Terms From Logistic Regression Models for the Subgroup Analysis eTable 5. Association Between Maternal Education Level and Double Burden of Malnutrition Subtypes in Different Income Groups (Odds Ratios [95% CIs]) eTable 6. Association Between Maternal Education Level and Double Burden of Malnutrition Subtypes in Different Income Groups, Further Adjusting for Paternal Education Level (Odds Ratios [95% CIs]) eTable 7. Association Between Maternal Education Level and Double Burden of Malnutrition Subtypes Without Using Multiple Imputation (Odds Ratios [95% CIs]) eTable 8. Association Between Maternal Education Level and Double Burden of Malnutrition Subtypes in Mothers With the Oldest Child (Odds Ratios [95% CIs]) eTable 9. Association Between Maternal Education Level and Double Burden of Malnutrition Subtypes, Further Excluding India (Odds Ratios [95% CIs]) eTable 10. Association Between Maternal Education Level and Double Burden of Malnutrition Subtypes, Without Adjusting for Birth Size (Odds Ratios [95% CIs]) eTable 11. Association Between Paternal Education Level and Double Burden of Malnutrition Subtypes, Further Adjusting for Maternal Education Level (Odds Ratios [95% CIs]) eTable 12. Association Between Paternal Education Level and Double Burden of Malnutrition Subtypes Without Using Multiple Imputation (Odds Ratios [95% CIs]) eTable 13. Association Between Paternal Education Level and Double Burden of Malnutrition Subtypes in Fathers With the Oldest Child (Odds Ratios [95% CIs]) eFigure 1. Flow Diagram Showing Exclusions and Final Sample Sizes of the Study Population in the Study of Mother-Child Pairs, Using the Most Recent Pooled Demographic and Household Surveys Data Since 2010 eFigure 2. Flow Diagram Showing Exclusions and Final Sample Sizes of the [file jamanetwopen-e2251727-s001.pdf]

Supplementary Online Content

Chen S, Richardson S, Kong Y, et al. Association between parental education and simultaneous malnutrition among parents and children in 45 low- and middle-income countries. *JAMA Netw Open*. 2023;6(1):e2251727. doi:10.1001/jamanetworkopen.2022.51727

eTable 1. Countries Included in the Analysis of Mother-Child Pairs

eTable 2. Countries Included in the Analysis of Father-Child Pairs

eTable 3. Definitions of Covariates

eTable 4. *P* Values of Tests of Interaction Terms From Logistic Regression Models for the Subgroup Analysis

eTable 5. Association Between Maternal Education Level and Double Burden of Malnutrition Subtypes in Different Income Groups (Odds Ratios [95% CIs])

eTable 6. Association Between Maternal Education Level and Double Burden of Malnutrition Subtypes in Different Income Groups, Further Adjusting for Paternal Education Level (Odds Ratios [95% CIs])

eTable 7. Association Between Maternal Education Level and Double Burden of Malnutrition Subtypes Without Using Multiple Imputation (Odds Ratios [95% CIs])

eTable 8. Association Between Maternal Education Level and Double Burden of Malnutrition Subtypes in Mothers With the Oldest Child (Odds Ratios [95% CIs])

eTable 9. Association Between Maternal Education Level and Double Burden of Malnutrition Subtypes, Further Excluding India (Odds Ratios [95% CIs])

eTable 10. Association Between Maternal Education Level and Double Burden of Malnutrition Subtypes, Without Adjusting for Birth Size (Odds Ratios [95% CIs])

eTable 11. Association Between Paternal Education Level and Double Burden of Malnutrition Subtypes, Further Adjusting for Maternal Education Level (Odds Ratios [95% CIs])

eTable 12. Association Between Paternal Education Level and Double Burden of Malnutrition Subtypes Without Using Multiple Imputation (Odds Ratios [95% CIs])

eTable 13. Association Between Paternal Education Level and Double Burden of Malnutrition Subtypes in Fathers With the Oldest Child (Odds Ratios [95% CIs])

eFigure 1. Flow Diagram Showing Exclusions and Final Sample Sizes of the Study Population in the Study of Mother-Child Pairs, Using the Most Recent Pooled Demographic and Household Surveys Data Since 2010

© 2023 Chen S et al. *JAMA Network Open.*

eFigure 2. Flow Diagram Showing Exclusions and Final Sample Sizes of the Study Population in the Study of Father-Child Pairs, Using the Most Recent Pooled Demographic and Household Surveys Data Since 2010

eFigure 3. Dose-Response Association Between Years of Maternal Education and Prevalence of Double Burden of Malnutrition

eFigure 4. Associations Between Maternal Education Level and Double Burden of Malnutrition Stratified by Child's Age, Sex, and Residence (Odds Ratios [95% CIs])

eFigure 5. Dose-Response Association Between Years of Paternal Education and Prevalence of Double Burden of Malnutrition Subtypes

eFigure 6. Associations Between Paternal Education Level and Double Burden of Malnutrition Subtypes Stratified by Child's Age, Sex, and Type of Residence (Odds Ratios [95% CIs])

This supplementary material has been provided by the authors to give readers additional information about their work.

© 2023 Chen S et al. *JAMA Network Open.*

eTable 1. Characteristics of Mother-Child Pairs

Country	Year	Income group	Number of Participants
Albania	2017-2018	Upper middle income	2,454
Armenia	2015-2016	Lower middle income	1,539
Benin	2017-2018	Low income	5,638
Burkina Faso	2010	Low income	6,161
Burundi	2016-2017	Low income	5,438
Cameroon	2018	Lower middle income	4,068
Chad	2014-2015	Low income	10,169
Comoros	2012	Low income	2,840
Congo	2011-2012	Lower middle income	4,192
Congo Democratic Republic	2013-2014	Low income	7,187
Cote d'Ivoire	2011-2012	Lower middle income	2,941
Dominican Republic	2013	Upper middle income	3,211
Gabon	2012	Upper middle income	3,155
Gambia	2019-2020	Low income	3,448
Ghana	2014	Lower middle income	2,490
Guatemala	2014-2015	Lower middle income	10,954
Guinea	2018	Low income	3,204
Haiti	2016-2017	Low income	3,507
Honduras	2011-2012	Lower middle income	9,683
India	2019-2021	Lower middle income	213,392
Jordan	2017-2018	Upper middle income	3,798
Kenya	2014	Lower middle income	9,068
Kyrgyz Republic	2012	Low income	3,699
Lesotho	2014	Lower middle income	1,309
Liberia	2019-2020	Low income	2,263
Malawi	2015-2016	Low income	4,907
Mali	2018	Low income	3,951
Mauritania	2019-2021	Lower middle income	4,793
Mozambique	2011	Low income	8,820
Myanmar	2015-2016	Lower middle income	4,021
Namibia	2013	Upper middle income	1,723
Nepal	2016	Low income	2,229
Niger	2012	Low income	4,846
Nigeria	2018	Lower middle income	10,002
Pakistan	2017-2018	Lower middle income	4,253
Rwanda	2019-2020	Low income	3,586
Sierra Leone	2019	Low income	3,901

© 2023 Chen S et al. *JAMA Network Open.*

eTable 1. Characteristics of Mother-Child Pairs (continued)

Country	Year	Income group	Number of Participants
South Africa	2016	Upper middle income	1,064
Tajikistan	2017	Low income	5,381
Tanzania	2015-2016	Low income	8,207
Timor-Leste	2016	Lower middle income	6,244
Togo	2014	Low income	2,914
Uganda	2016	Low income	3,926
Zambia	2018	Lower middle income	8,031
Zimbabwe	2015	Low income	4,733

© 2023 Chen S et al. *JAMA Network Open.*

eTable 2. Characteristics of Father-Child Pairs

Country	Year	Income group	Number of Participants
Albania	2017-2018	Upper middle income	881
Burkina Faso	2010	Low income	5,252
Congo Democratic Republic	2013-2014	Low income	5,543
Cote d'Ivoire	2011-2012	Lower middle income	1,819
Gabon	2012	Upper middle income	1,620
India	2019-2021	Lower middle income	23,960
Lesotho	2014	Lower middle income	417
Namibia	2013	Upper middle income	618
Nepal	2016	Low income	1,212
Niger	2012	Low income	3,195
Sierra Leone	2019	Low income	2,503
South Africa	2016	Upper middle income	246
Timor-Leste	2016	Lower middle income	1,825
Togo	2014	Low income	2,192
Uganda	2016	Low income	2,750
Zimbabwe	2015	Low income	2,687

© 2023 Chen S et al. *JAMA Network Open.*

eTable 3. Definitions of Covariates

Risk factor	Definition	Reference category	Category type
Child factors			
Child's sex	The sex of the child. In the 2 following categories: (1) male; (2) female.	Male	Binary variable
Child birth size	The birth size of child. according to mother's estimate of baby's size at time of birth, it is divided into the 3 following categories: (1) smaller than average; (2) average, and (3) bigger than average.	Average	Categorical variable
Household factors			
Type of residence	According to the place where the households live, it is divided into the 2 following categories:(1) urban; (2) rural	Urban	Binary variable
Household wealth quintile	According to a selected set of household assets in 5 quintiles constructed by DHS, it is divided into the 5 following categories: (1) poorest household wealth; (2) poorer; (3) middle; (4) richer;(5) richest	Richest	Categorical variable
Sex of the household head	According to the sex of the head of household, it is divided into the 2 following categories: (1) male; (2) female.	Male	Binary variable
Environmental factors			
Indoor pollution	According to the fuel used in the household, it is divided into the 2 following categories: (1) low: use solid fuels for cooking; (2) high: use other kinds of fuels	Low indoor pollution	Binary variable
Drinking water source	According to how the household get their drinking water, it is divided into the 2 following categories: (1) safe: use water piped, tube well or borehole, public tap or standpipe, protected well or spring, and bottled water; (2) unsafe: use other kinds of ways to get water.	Safe water	Binary variable
Sanitary facility	According to the type of latrine the household uses, it is divided into the 2 following categories: (1) improved: the household use flush to piped sewer system, ventilated improved pit latrine, septic tank or pit latrine, composting toilet, and pit latrine with slab; (2) unimproved: use other kinds of ways to g dispose of feces.	Improved sanitation facility	Binary variable

© 2023 Chen S et al. *JAMA Network Open.*

eTable 3. Definitions of Covariates (continued)

**Risk factor**	**Definition**	**Reference category**	**Category type**
Parental factors			
Skilled birth attendant at delivery	According to whether the woman has a skilled birth attendant when delivered the child, it is divided into the 2 following categories: (1) Yes: with skilled birth attendant, including physicians, nurses; (2) no: others.	Yes	Binary variable
Family planning needs	According to the family planning needs of women, it is divided into the 2 following categories: (1) satisfied: the fecund and married women wish to stop or delay childbearing or have accessing to any modern method of contraception; (2) unsatisfied otherwise	Satisfied	Binary variable
Maternal age at marriage	According to the mother's age at the time of marriage, it is divided into the 2 following categories: (1) child marriage: married at <18 y and (2) no: others.	No	Binary variable
Paternal age at marriage	According to the father's age at the time of marriage, it is divided into the 2 following categories: (1) child marriage: married at <18 y and (2) no: others.	No	Binary variable

© 2023 Chen S et al. *JAMA Network Open.*

eTable 4. *P* Values of Tests of Interaction Terms From Logistic Regression Models for the Subgroup Analysis

	DBM in mother-child pairs	Maternal overnutrition and child undernutrition	Maternal undernutrition and child overnutrition	Maternal overnutrition and child overnutrition	Maternal undernutrition and child undernutrition
Maternal education × children's age	<0.001	0.025	<0.001	0<0.001	0.004
Maternal education × children's sex	0.920	0.913	0.001	0.788	0.980
Maternal education × residence	0.457	0.001	0.440	0.902	0.785

© 2023 Chen S et al. *JAMA Network Open.*

eTable 5. Association Between Maternal Education Level and Double Burden of Malnutrition Subtypes in Different Income Groups (Odds Ratios [95% CIs])

	Income Groups		
	**Low income**	**Lower middle income**	**Upper middle income**
**Number of countries**	20	19	6
**Double burden of malnutrition in mother-child pairs**			
No education	1.00 [Reference]	1.00 [Reference]	1.00 [Reference]
Primary education	0.88 (0.82, 0.93)	0.95 (0.91, 1.00)	1.20 (0.58, 2.50)
Secondary education	0.82 (0.76, 0.88)	0.90 (0.86, 0.93)	1.33 (0.67, 2.62)
Tertiary education	0.62 (0.55, 0.71)	0.72 (0.68, 0.77)	1.09 (0.52, 2.29)
**Maternal overnutrition and child undernutrition**			
No education	1.00 [Reference]	1.00 [Reference]	1.00 [Reference]
Primary education	1.13 (1.02, 1.25)	1.25 (1.17, 1.34)	1.77 (0.70, 4.51)
Secondary education	1.18 (1.05, 1.32)	1.34 (1.26, 1.43)	1.93 (0.82, 4.55)
Tertiary education	0.88 (0.74, 1.04)	1.35 (1.25, 1.46)	1.54 (0.61, 3.91)
**Maternal undernutrition and child overnutrition**			
No education	1.00 [Reference]	1.00 [Reference]	1.00 [Reference]
Primary education	1.23 (0.87, 1.74)	0.74 (0.57, 0.95)	1.56 (0.24, 10.13)
Secondary education	1.88 (1.21, 2.94)	1.07 (0.89, 1.29)	1.56 (0.23, 10.42)
Tertiary education	1.56 (0.80, 3.03)	1.10 (0.84, 1.43)	2.01 (0.25, 16.14)
**Maternal overnutrition and child overnutrition**			
No education	1.00 [Reference]	1.00 [Reference]	1.00 [Reference]
Primary education	1.39 (0.85, 2.27)	1.68 (1.07, 2.66)	3.81 (0.92, 15.72)
Secondary education	1.56 (0.90, 2.69)	2.19 (1.50, 3.19)	4.48 (1.08, 18.53)
Tertiary education	1.59 (0.80, 3.17)	3.21 (2.06, 5.01)	4.35 (1.00, 18.88)
**Maternal undernutrition and child undernutrition**			
No education	1.00 [Reference]	1.00 [Reference]	1.00 [Reference]
Primary education	0.86 (0.81, 0.92)	0.93 (0.89, 0.97)	1.03 (0.49, 2.16)
Secondary education	0.78 (0.72, 0.85)	0.83 (0.80, 0.87)	0.91 (0.45, 1.81)
Tertiary education	0.61 (0.53, 0.71)	0.65 (0.61, 0.69)	0.84 (0.35, 2.00)

© 2023 Chen S et al. *JAMA Network Open.*

eTable 6. Association Between Maternal Education Level and Double Burden of Malnutrition Subtypes in Different Income Groups, Further Adjusting for Paternal Education Level (Odds Ratios [95% CIs])

	**DBM in mother-child pairs**	**Maternal overnutrition and child undernutrition**	**Maternal undernutrition and child overnutrition**	**Maternal overnutrition and child overnutrition**	**Maternal undernutrition and child undernutrition**
No education	1.00 [Reference]	1.00 [Reference]	1.00 [Reference]	1.00 [Reference]	1.00 [Reference]
Primary education	0.94 (0.89, 0.99)	1.15 (1.06, 1.26)	0.93 (0.66, 1.30)	2.05 (1.24, 3.38)	0.91 (0.86, 0.97)
Secondary education	0.88 (0.83, 0.94)	1.16 (1.05, 1.28)	1.14 (0.79, 1.64)	2.55 (1.46, 4.45)	0.81 (0.75, 0.86)
Tertiary education	0.68 (0.61, 0.75)	1.01 (0.88, 1.17)	0.77 (0.42, 1.41)	2.74 (1.41, 5.33)	0.65 (0.58, 0.72)

Abbreviations: DBM, double burden of malnutrition.

Note: The model was adjusted for country-fixed effects, child’s age, child’s birth order, mother’s age, child’s sex, child birth size, type of residence, household wealth quintile, skilled birth attendant at delivery, family planning needs satisfied, maternal age at marriage, the sex of household head, indoor pollution, indoor pollution, drinking water source, sanitary facility, and paternal education.

© 2023 Chen S et al. *JAMA Network Open.*

eTable 7. Association Between Maternal Education Level and Double Burden of Malnutrition Subtypes Without Using Multiple Imputation (Odds Ratios [95% CIs])

	**DBM in mother-child pairs**	**Maternal overnutrition and child undernutrition**	**Maternal undernutrition and child overnutrition**	**Maternal overnutrition and child overnutrition**	**Maternal undernutrition and child undernutrition**
No education	1.00 [Reference]	1.00 [Reference]	1.00 [Reference]	1.00 [Reference]	1.00 [Reference]
Primary education	0.95 (0.91, 0.98)	1.22 (1.15, 1.30)	0.78 (0.62, 0.99)	1.72 (1.20, 2.47)	0.92 (0.89, 0.96)
Secondary education	0.89 (0.86, 0.93)	1.32 (1.25, 1.40)	1.10 (0.91, 1.32)	2.09 (1.52, 2.87)	0.84 (0.81, 0.87)
Tertiary education	0.72 (0.68, 0.76)	1.30 (1.20, 1.40)	1.05 (0.80, 1.36)	2.54 (1.73, 3.73)	0.66 (0.62, 0.70)

Abbreviations: DBM, double burden of malnutrition.

© 2023 Chen S et al. *JAMA Network Open.*

eTable 8. Association Between Maternal Education Level and Double Burden of Malnutrition Subtypes in Mothers With the Oldest Child (Odds Ratios [95% CIs])

	**DBM in mother-child pairs**	**Maternal overnutrition and child undernutrition**	**Maternal undernutrition and child overnutrition**	**Maternal overnutrition and child overnutrition**	**Maternal undernutrition and child undernutrition**
No education	1.00 [Reference]	1.00 [Reference]	1.00 [Reference]	1.00 [Reference]	1.00 [Reference]
Primary education	0.94 (0.90, 0.98)	1.28 (1.20, 1.37)	0.75 (0.54, 1.04)	1.53 (0.95, 2.47)	0.91 (0.87, 0.95)
Secondary education	0.88 (0.85, 0.91)	1.38 (1.29, 1.46)	1.23 (0.97, 1.56)	2.16 (1.42, 3.27)	0.82 (0.79, 0.85)
Tertiary education	0.71 (0.67, 0.75)	1.37 (1.26, 1.48)	1.13 (0.83, 1.55)	2.48 (1.56, 3.95)	0.65 (0.61, 0.69)

Abbreviations: DBM, double burden of malnutrition.

© 2023 Chen S et al. *JAMA Network Open.*

eTable 9. Association Between Maternal Education Level and Double Burden of Malnutrition Subtypes, Further Excluding India (Odds Ratios [95% CIs])

	DBM in mother-child pairs	Maternal overnutrition and child undernutrition	Maternal undernutrition and child overnutrition	Maternal overnutrition and child overnutrition	Maternal undernutrition and child undernutrition
No education	1.00 [Reference]	1.00 [Reference]	1.00 [Reference]	1.00 [Reference]	1.00 [Reference]
Primary education	0.89 (0.84, 0.94)	1.24 (1.13, 1.36)	0.87 (0.64, 1.19)	1.68 (1.08, 2.62)	0.83 (0.79, 0.88)
Secondary education	0.83 (0.77, 0.89)	1.26 (1.13, 1.41)	1.33 (0.91, 1.95)	2.21 (1.41, 3.47)	0.72 (0.67, 0.78)
Tertiary education	0.64 (0.57, 0.72)	0.91 (0.77, 1.06)	1.10 (0.60, 2.01)	2.69 (1.49, 4.86)	0.59 (0.52, 0.68)

Abbreviations: DBM, double burden of malnutrition.

© 2023 Chen S et al. *JAMA Network Open.*

eTable 10. Association Between Maternal Education Level and Double Burden of Malnutrition Subtypes, Without Adjusting for Birth Size (Odds Ratios [95% CIs])

	DBM in mother-child pairs	Maternal overnutrition and child undernutrition	Maternal undernutrition and child overnutrition	Maternal overnutrition and child overnutrition	Maternal undernutrition and child undernutrition
No education	1.00 [Reference]	1.00 [Reference]	1.00 [Reference]	1.00 [Reference]	1.00 [Reference]
Primary education	0.94 (0.90, 0.97)	1.26 (1.19, 1.34)	0.79 (0.63, 0.98)	1.77 (1.25, 2.50)	0.91 (0.88, 0.95)
Secondary education	0.89 (0.86, 0.92)	1.35 (1.28, 1.43)	1.12 (0.94, 1.34)	2.24 (1.64, 3.06)	0.83 (0.80, 0.86)
Tertiary education	0.70 (0.67, 0.74)	1.33 (1.24, 1.43)	1.13 (0.88, 1.46)	3.05 (2.09, 4.45)	0.64 (0.61, 0.68)

Abbreviations: DBM, double burden of malnutrition.

© 2023 Chen S et al. *JAMA Network Open.*

eTable 11. Association Between Paternal Education Level and Double Burden of Malnutrition Subtypes, Further Adjusting for Maternal Education Level (Odds Ratios [95% CIs])

	DBM in father-child pairs	Paternal overnutrition and child undernutrition	Paternal undernutrition and child undernutrition
No education	1.00 [Reference]	1.00 [Reference]	1.00 [Reference]
Primary education	0.89 (0.78, 1.02)	1.12 (0.89, 1.41)	0.85 (0.74, 0.97)
Secondary education	0.98 (0.86, 1.10)	1.33 (1.08, 1.63)	0.88 (0.77, 1.00)
Tertiary education	1.01 (0.86, 1.19)	1.46 (1.14, 1.87)	0.81 (0.68, 0.98)

Abbreviations: DBM, double burden of malnutrition.

© 2023 Chen S et al. *JAMA Network Open.*

eTable 12. Association Between Paternal Education Level and Double Burden of Malnutrition Subtypes Without Using Multiple Imputation (Odds Ratios [95% CIs])

	**DBM in father-child pairs**	**Paternal overnutrition and child undernutrition**	**Paternal undernutrition and child undernutrition**
No education	1.00 [Reference]	1.00 [Reference]	1.00 [Reference]
Primary education	0.90 (0.79, 1.03)	1.13 (0.89, 1.43)	0.85 (0.74, 0.98)
Secondary education	0.98 (0.86, 1.11)	1.32 (1.07, 1.63)	0.88 (0.77, 1.00)
Tertiary education	1.00 (0.85, 1.18)	1.47 (1.14, 1.89)	0.80 (0.66, 0.96)

Abbreviations: DBM, double burden of malnutrition.

© 2023 Chen S et al. *JAMA Network Open.*

eTable 13. Association Between Paternal Education Level and Double Burden of Malnutrition Subtypes in Fathers With the Oldest Child (Odds Ratios [95% CIs])

	**DBM in father-child pairs**	**Paternal overnutrition and child undernutrition**	**Paternal undernutrition and child undernutrition**
No education	1.00 [Reference]	1.00 [Reference]	1.00 [Reference]
Primary education	0.88 (0.77, 1.00)	1.13 (0.89, 1.44)	0.85 (0.74, 0.97)
Secondary education	0.92 (0.81, 1.04)	1.33 (1.09, 1.63)	0.81 (0.72, 0.92)
Tertiary education	0.85 (0.73, 1.01)	1.45 (1.14, 1.84)	0.67 (0.56, 0.80)

Abbreviations: DBM, double burden of malnutrition.

© 2023 Chen S et al. *JAMA Network Open.*

eFigure 1. Flow Diagram Showing Exclusions and Final Sample Sizes of the Study Population in the Study of Mother-Child Pairs, Using the Most Recent Pooled Demographic and Household Surveys Data Since 2010

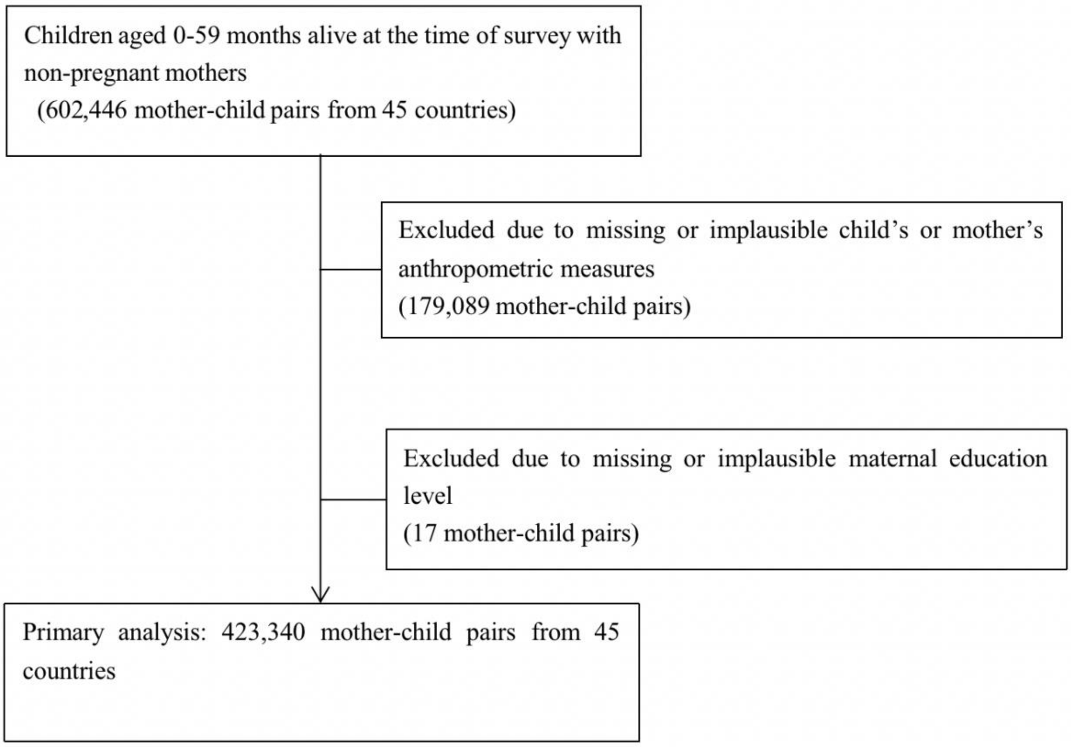

eFigure 2. Flow Diagram Showing Exclusions and Final Sample Sizes of the Study Population in the Study of Father-Child Pairs, Using the Most Recent Pooled Demographic and Household Surveys Data Since 2010

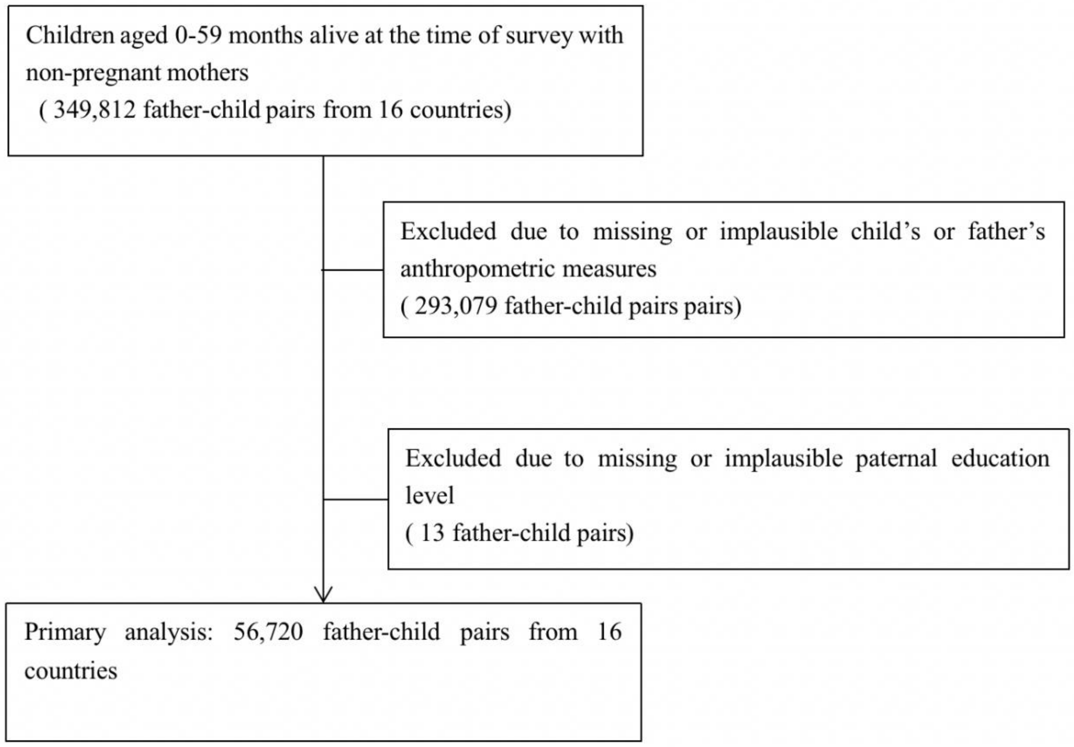

© 2023 Chen S et al. *JAMA Network Open.*

eFigure 3. Dose-Response Association Between Years of Maternal Education and Prevalence of Double Burden of Malnutrition

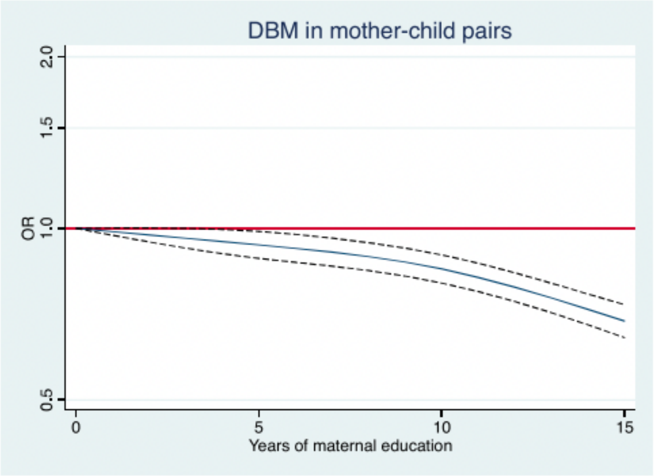

© 2023 Chen S et al. *JAMA Network Open.*

eFigure 4. Associations Between Maternal Education Level and Double Burden of Malnutrition Stratified by Child's Age, Sex, and Residence (Odds Ratios [95% CIs])

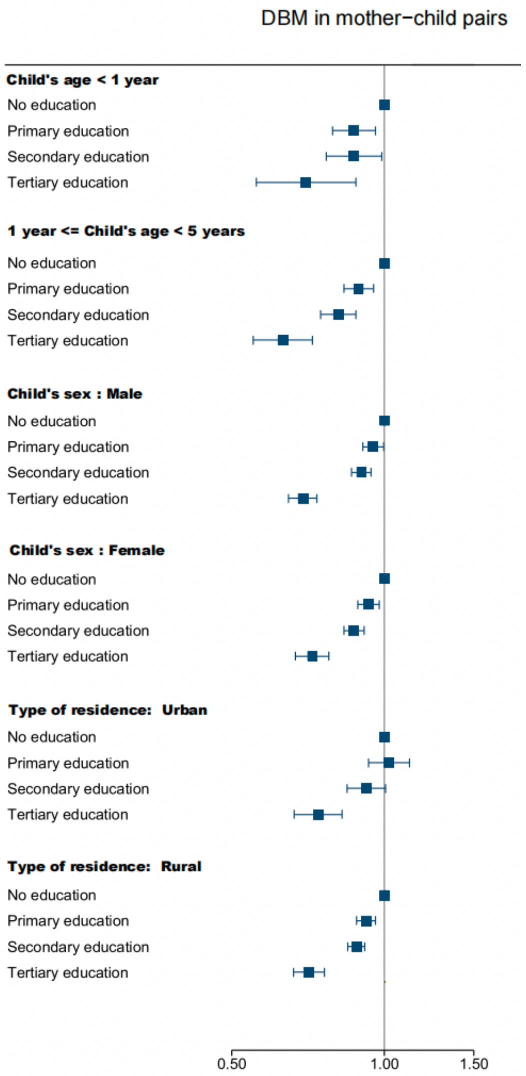

© 2023 Chen S et al. *JAMA Network Open.*

eFigure 5. Dose-Response Association Between Years of Paternal Education and Prevalence of Double Burden of Malnutrition Subtypes

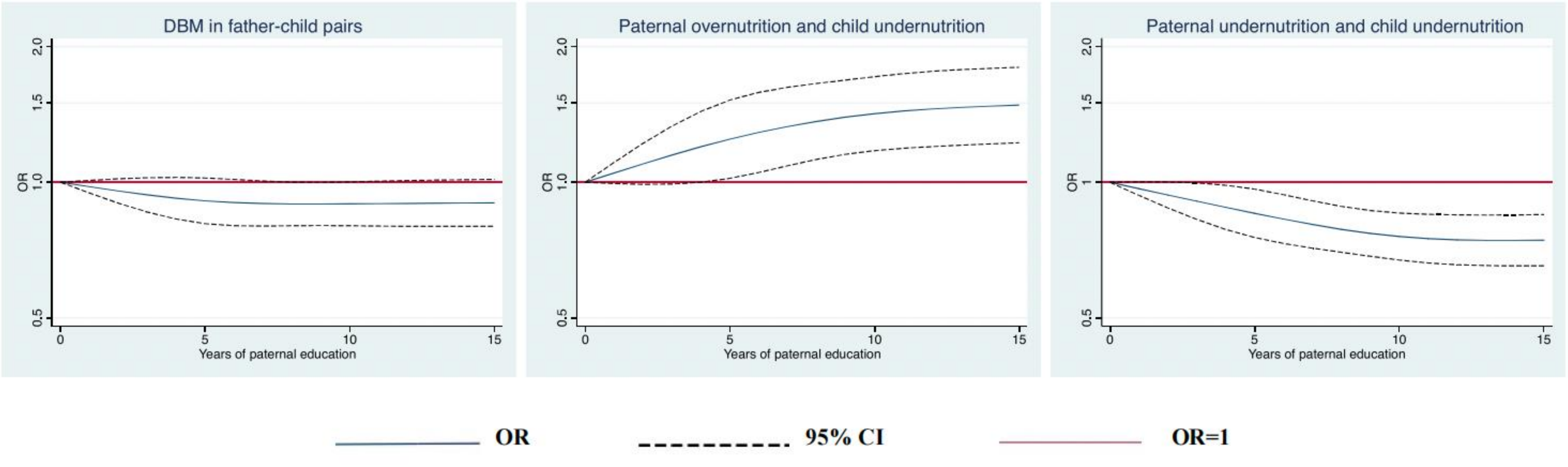

© 2023 Chen S et al. *JAMA Network Open.*

eFigure 6. Associations Between Paternal Education Level and Double Burden of Malnutrition Subtypes Stratified by Child's Age, Sex, and Type of Residence (Odds Ratios [95% CIs])

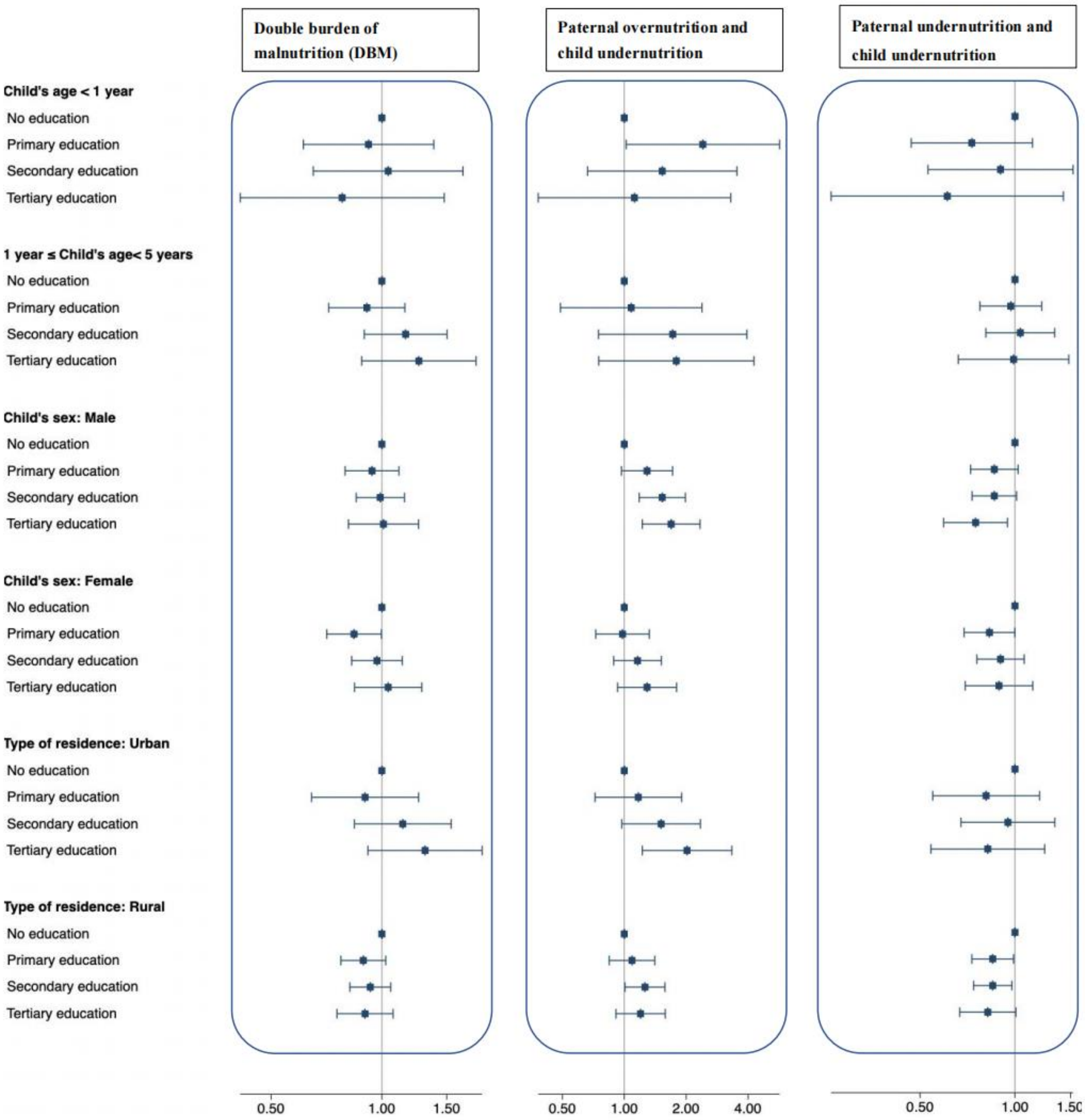

© 2023 Chen S et al. *JAMA Network Open.*
