# Supplementary material for: Association Between Parental Education and Simultaneous Malnutrition Among Parents and Children in 45 Low- and Middle-Income Countries
Source: JAMA Netw Open. 2023 Jan 24;6(1):e2251727. doi: 10.1001/jamanetworkopen.2022.51727 (PMC10408270; doi:10.1001/jamanetworkopen.2022.51727)
Supplement: Supplement 2. — Data Sharing Statement [file jamanetwopen-e2251727-s002.pdf]

Data Sharing Statement

Chen. Association Between Parental Education and Simultaneous Malnutrition Among Parents and Children in 45 Low- and Middle-Income Countries. *JAMA Netw Open*. Published January 24, 2023. doi:10.1001/jamanetworkopen.2022.51727

Data
Data available: Yes
Data types: Deidentified participant data
How to access data: zhihuili@tsinghua.edu.cn
When available: With publication

Supporting Documents
Document types: None

Additional Information
Who can access the data: researchers whose proposed use of the data has been approved
Types of analyses: for a specified purpose
Mechanisms of data availability: The data set used in this study can be accessed free of charge from Ahinkorah BO, Amadu I, Seidu AA, et al. Prevalence and factors associated with the triple burden of malnutrition among mother-child pairs in sub-Saharan Africa. *Nutrients*. 2021;13(6):2050. https://doi.org/10.3390/nu13062050
